# Mini-Galaxy: Rethinking Complex Human Diseases Through the Lens of Systems Biology and Multilayered AI Network Perspectives

**DOI:** 10.3390/ijms27073161

**Published:** 2026-03-31

**Authors:** Cristina Correia, Choong Yong Ung, Zhuofei Zhang, Easton Blissenbach, Shizhen Zhu, Daniel D. Billadeau, Yuin-Han Loh, Hu Li

**Affiliations:** 1 Department of Molecular Pharmacology and Experimental Therapeutics, Mayo Clinic College of Medicine and Science, Rochester, MN 55905, USA; 2 Bioinformatics and Computational Biology, University of Minnesota, Rochester, MN 55905, USA; 3 Department of Biochemistry and Molecular Biology, Mayo Clinic College of Medicine and Science, Rochester, MN 55905, USA; 4 Department of Immunology, Mayo Clinic College of Medicine and Science, Rochester, MN 55905, USA; 5 Epigenetics and Cell Fates Laboratory, Cell Fate Engineering and Therapeutics Laboratory, Institute of Molecular and Cell Biology (IMCB), Agency for Science, Technology and Research (A*STAR), 61 Biopolis Drive Proteos, Singapore 138673, Singapore; 6 Department of Biological Sciences, Faculty of Science, National University of Singapore, 14 Science Drive 4, Singapore 117543, Singapore; 7 Department of Physiology, NUS Yong Loo Lin School of Medicine, 2 Medical Drive, MD9, Singapore 117593, Singapore; 8 Division of Gastroenterology and Hepatology, Department of Medicine, Mayo Clinic, Rochester, MN 55905, USA; 9 Paul F. Glenn Center for Biology of Aging Research, Mayo Clinic, Rochester, MN 55905, USA

**Keywords:** systems biology, artificial intelligence, network, salient gene properties, latent gene properties

## Abstract

Human diseases are complex and arise from the coordinated action of multiple genes and their protein products. Genes’ behaviors extend beyond genetic variants, mutations, and differential expressions. Their coordinated activity across biological scales (molecules, cells, tissues, organs) produces emergent behaviors that shape health and disease. These emergent behaviors span time and space and are often hard to measure directly from observation when using standard experimental measurements. Yet these “hidden” or latent gene characteristics can be powerful drivers of disease. We propose a Mini-Galaxy Model (MGM), a systems-level AI-driven network framework that models cells as “mini-galaxies” composed of multilayered biological information, with each layer encoding a different dimension of genes’ behavior. Here, we delineate a strategy on how to construct and compare MGMs across health and disease and map their etiological relatedness. We also operationalize the MGM as a discovery platform for translational medicine, offering modules to allow target prioritization and editing. By reframing human diseases as the result of emergent behavior of multilayered multimode biological networks and their perturbations, the MGM yields actionable rules to streamline biomarker discovery, guide target selection and enable rational design of combinatorial interventions, and accelerate drug repurposing.

## 1. Introduction

Human complex diseases are inherently multi-factorial and multi-scale. Disease phenotypes emerge from the interplay of many genes, dynamic cell–cell communications, and cross-level processes that span from molecular, cellular, tissue, organ, and organismal scales [1,2,3]. Classical gene- and pathway-centric strategies struggle to account for such system complexity. In most disease conditions, there is no single gene, nor do recurrent genetic mutations or epigenetic alterations suffice to explain disease onset and progression. Rather, a constellation of genes acts in concert to drive and sustain disease. Moreover, differentially expressed genes are often not the primary drivers of disease progression [4].

Network biology reframes these observations by perceiving cellular processes as networks of interconnected entities like genes, proteins, metabolites, and others. Disease interactomes can then be distilled, mainly into protein–protein interactions (PPIs). Under this network framework, a disease results from the perturbation of biological interconnected modules rather than isolated “islands” of genetic defects [5,6]. Empirical analyses show that genes contributing to the same disease cluster are located within specific neighborhoods of the PPI network, and the proximities and overlaps among disease modules can help explain relationships among diseases [7]. These network perspectives shift the focus from single genes to the misorchestration of gene groups within the cellular interactome.

Our earlier studies using state-of-the-art systems biology and Artificial Intelligence (AI) approaches reveal that genes display an array of traits, including both observable (salient) features and latent properties, the latter being inaccessible to standard bioinformatics and statistical approaches. Latent properties include, for example, the concept of gene utility [8], where a genes’s importance is defined by its role within a network and its interactions with neighboring genes, reflecting the amount of information flow within a network. Rheostat-like associations that describe genes acting as genetic switches that can govern the transition between different biological contexts [9], “dark” associations that do not involve mutated and differentially expressed genes [10], and invariant gene pairs, whose combined expression is invariant across individuals of the same biological phenotype regardless of their genetic heterogeneity [11] are other examples of latent properties of genes. Unlike genetic mutations and differential expressions, these gene-latent characteristics are inferred as they cannot be observed and measured directly. Each type of latent characteristic represents a different layer of biological information that can fill the void in our current understanding. Integrative analyses on these multiple layers of biological information can yield novel insights into disease initiation and progression and inform the design of more effective therapies.

**Figure 1:**
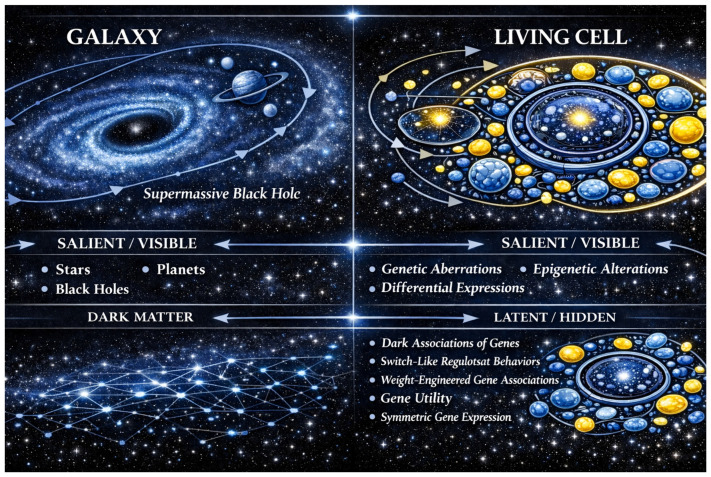
The Mini-Galaxy framework. Similar to a galaxy that exhibits both salient (observable solar and planetary objects) and hidden entities (e.g., black holes and dark matter), these components coalesce into a unified system. Analogously, cellular phenotypes arise from the collective action of gene networks and their salient and latent properties. A cell is viewed as a functional multimode network, like a “mini-galaxy”, where each gene’s behavior is shaped by the combined actions of salient and latent properties. Salient gene properties (e.g., genetic mutations, differential expressions, etc.) can be observed and measured directly, whereas latent gene properties (e.g., gene utility, symmetric gene expression, etc.) cannot be observed directly and must be inferred through hypothesis-driven computational approaches. The number of latent properties is currently unknown. (Figure generated by ChatGPT GPTv5.3.).

In this perspective article, we introduce the Mini-Galaxy Model (MGM) as a conceptual and operational scaffold for representing complex and multilayered cellular regulation. Just like a galaxy that possesses both observational and hidden parts, a biological network can also encode both salient and latent gene characteristics. For example, within galaxies, there is a black hole that spurs the connectiveness of the system. There is also evidence that within galaxies the remains of ancient stars exist; however, these are rarely seen or measured. We therefore use a galaxy as a metaphor to emphasize the interconnectedness of gene-salient and -latent properties in mirroring the intricate multi-scale dynamics of genes and cellular regulation, where genes carry information across scales (Figure 1). Our Mini-Galaxy Model is a conceptual and operational scaffold for understanding how genes exert multimodal actions. The MGM treats a cell as a mini-galaxy composed of multi-dimensional interactomes. Within the MGM framework, a disease emerges as the systems-level outcome of multiple defects arising from different information layers. Genes that act across several informational layers are nominated as multi-dimensional information hubs, akin to gravitational centers within a galaxy. Operationally, the MGM provides a unifying language for integrating both salient and a suite of latent genetic characteristics that collectively shape a disease phenotype.

The concept of the MGM is grounded in network biology and medicine, and multilayered network paradigms. The novelty of the MGM lies in its explicit and interpretable scaffold that organizes hypothesis-guided salient gene and latent gene properties into structured layers that are enriched in integrated network biology semantics. To our knowledge, this multimode network has not been clearly articulated or systematically established. As a starting point, we first present the MGM conceptual framework and define what we mean by salient versus latent gene properties. We then outline strategies for constructing a disease-specific MGM and for nominating critical information hubs that integrate signals across layers. Finally, we discuss how the MGM can serve as a discovery platform for translational medicine.

## 2. Mini-Galaxy Model (MGM)

The central tenet of the Mini-Galaxy Model (MGM) is that each living cell is perceived as a “galaxy”. Both living cells and galaxies consist of networks of interactions that give rise to self-organized systems. In a galaxy, stars, planets, black holes, and dark matter are bound together through gravitational forces, forming a spiral-armed structure that is stabilized via gravitational feedback among solar systems and a huge gravitational center (that is believed to be a gigantic black hole). Similarly, within a cell, DNA, RNA, proteins, and biomolecular complexes interact through biochemical forces and signaling networks, generating stable yet adaptable subnetwork coordination that safeguards cellular homeostasis. A galaxy is composed of salient (visible and directly observable) components such as stars and planets, and latent (hidden or inferred) components, such as black holes and dark matter, act as the invisible scaffold of the universe, whose interplay determines its structure’s growth and evolution. Similarly, a cell is composed of both salient (e.g., genetic aberrations, epigenetic alterations, and differential expressions) and latent (e.g., dark associations of genes, as well as switch-like regulostat behavior, weight-engineered gene associations, gene utility, and symmetric gene expression) gene properties that sustain the phenotypic behavior of a cell, including disease.

The MGM is distinct from existing biological network models in that it is a multimode gene network model that integrates both salient and latent gene properties to cohesively build a system and thus resembles a “mini-galaxy” (Figure 1). Also, unlike medical knowledge graphs that use measurable data attributes as nodes (i.e., data attributes) and edges (i.e., types of associations between different data attributes) derived from sources like genomics, transcriptomics, medical texts, and images [12], the MGM offers a distinct approach by using phenotypic data (e.g., transcriptomics) while aggregating various latent gene characteristics that cannot be directly observed or measured. Hence, the MGM is a hybrid, multimode and multimodal model, with each element of the MGM playing a crucial role in the integration and reconstruction of disease mechanisms.

**Figure 2:**
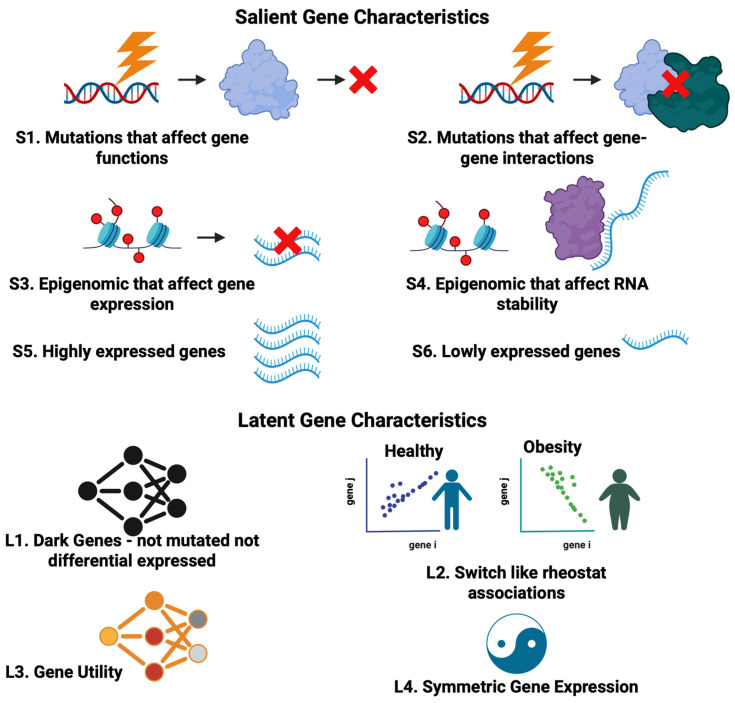
Distinct mechanisms of gene actions. Genes exhibit different “facets” that reflect either their salient or latent properties. Salient gene properties are directly observed and measured via experimental approaches. These include genetic mutations and variants that affect gene function or gene–gene interactions, epigenetic modifications that impact gene expression and the stability of RNA transcripts, as well as differential gene expression across biological contexts. In contrast, latent gene properties can only be inferred through computational approaches that are designed based on predefined concepts or hypotheses. These include, but are not restricted to, dark gene associations, switch-like regulostat gene–gene associations, gene utility, and invariant gene expressions. (Created in BioRender. Li, H. (2026) https://BioRender.com/p8sraui).

In this model, genes that anchor multiple other genes across both latent and salient information layers are particularly important, functioning as the galaxy’s “gravitational” centers. These informational hubs play key roles in disease and may be viable therapeutic targets. With the MGM, our goal is to pinpoint a concise set of latent genetic variables that encapsulate the action of gene products, such as functional mutations and relationships between gene expression, that are influential across different biological information layers. Here, we use the word galaxy as a metaphor to emphasize multimode interconnectedness and any hidden influences (e.g., dark associations), with the MGM represented as a multilayer gene network. This metaphor is intended to be illustrative rather than literal, and the key methodological contributions lie in how diverse salient and latent gene properties are defined, integrated, and interrogated within multilayered networks. Below, we describe in more detail the salient and latent gene properties that make up the MGM (Figure 2).

### 2.1. Salient Gene Properties

Salient gene properties are genetic properties that can be measured and observed directly via experimental and statistical approaches. These include genetic aberrations, epigenetic modifications, and altered gene expression (Figure 2, top). Since these salient gene properties are well-covered in the literature, we only provide a succinct description here. Our focus is on the latent properties described in the sections below.

#### 2.1.1. Genomic Aberrations

In general, genetic alterations include inheritable germline variants [13] that can cause Mendelian diseases or increase the risk of developing common diseases such as metabolic syndromes, Alzheimer’s disease, and nonheritable somatic mutations that are acquired over one’s lifetime [14], including acquiring somatic mutations in specific cell lineages that contribute to the development of various tissue-specific diseases, especially cancers [15]. However, many genetic mutations are either highly deleterious, causing cell death before they can cause disease, or they exert effects that are so small that no single mutation is sufficient to initiate disease onset. To address this, Castro-Giner et al. proposed the “mini-drivers” model, where, instead of acting alone, genetic mutations act together as “mini-drivers” to drive disease onset and progression [4].

#### 2.1.2. Epigenetic Modifications and Gene Expression Regulation

Epigenetic modifications broadly govern gene activation. These include DNA methylation [16] and histone modifications [17] that inform cells on the types of chromatin regions that should be actively transcribed and which regions should be silenced. MicroRNAs [18] and RNA-binding proteins [19] represent additional layers of regulation that modulate the stability of RNA transcripts and impact gene expression.

#### 2.1.3. Differential Gene Expression

Finding differences in gene expression is a key step in data analyses to compare how two biological states are distinct. Identifying differentially expressed genes not only allows us to detect which genes are upregulated or downregulated, but also permits researchers to uncover which biological pathways or processes play important roles in a certain biological state [20]. Identifying differentially expressed genes and corresponding pathways has been instrumental to our understanding of diseases [21] and drives biomarker discovery [22].

### 2.2. Latent Gene Properties

Current research often overlooks the multifaceted behavior of genes and how different gene properties layers interact to collectively contribute to disease. This gap is evident in the lack of conceptual frameworks and algorithmic strategies aiming to identify these latent properties. Although other computational frameworks used diffusion-based prioritization, disease-module expansion, differential co-expression, and interpretable machine learning representations as ways to extract latent attributes [23,24,25,26], there remains a lack of conceptual scaffolds for organizing these disparate layers into a unified network representation. Next, we highlight a set of latent gene properties—the “dark matter” of a living cell—that we and others have previously investigated (Figure 2, bottom) while reiterating that this set is not exhaustive and will likely expand with future discoveries.

In this context, the latent gene properties mentioned are distinct from latent embeddings generated from deep neural networks, with the latter referring to compressed, numerical vector representations that encode information from complex input data but often lack direct biological interpretability. In contrast, the latent properties we propose are hypothesis-guided and biologically grounded constructs and are not purely mathematic abstractions. We anticipate that this perspective will encourage the scientific community to more deeply investigate latent gene characteristics and develop novel approaches to enrich the MGMs described in this work.

#### 2.2.1. Dark Associations of Genes

We previously developed the Machine Learning-Assisted Network Inference (MALANI) method to reverse engineer networks that consist of “dark” or class II disease-associated genes [10]. The underlying premise is that, at the network scale, some genes that are neither differentially expressed nor mutated can still be critical for completing signal transmission paths and can be used to effectively “connect the dots” between canonical players and downstream genes that execute the response. MALANI employs a dot product, a linear algebra operation that multiplies corresponding elements of two equal-length gene expression vectors (from two genes across samples) and sums the results to produce a single scalar value that summarizes their joint pattern. These scalar dot products are then used as features in machine learning models to distinguish cancer from normal samples. In this proof-of-principle study, we used transcriptomics data across nine cancer types. We employed a support vector machine (SVM) to train SVM models by using dot products of gene pairs as inputs. More than 2 × 10^8^ SVM models were constructed for each cancer type. Gene pairs whose expression dot products improved classification performance (cancer vs. normal control samples) were deemed biologically relevant in cancer. These gene pairs were then assembled into SVM-inferred cancer-specific networks. Our study revealed that ~3% of genes in the genome are “dark” cancer genes because these are neither mutated nor differentially expressed genes, and thus frequently escape detection by conventional bioinformatics analyses. We also found that highly mutated oncogenes are frequently found in the shortest paths that connect gene pairs in the SVM-inferred network, which indicates strong functional associations between dark genes and mutated genes.

This study was pioneering in showing that machine learning (ML) can be employed to uncover hidden gene properties that do not conform to classic behaviors (i.e., no recurrent mutation and not differentially expressed) but can be influential by linking to certain mutated and dysregulated genes in disease. These nonlinear patterns of dark gene associations cannot be captured via conventional statistical approaches; however, machine learning approaches have the power to detect them.

#### 2.2.2. Switch-like Regulostat Behavior

Biological phenotypes, such as disease susceptibility or drug sensitivity, result from the complex interplay of a myriad of genes. Rather than behaving like on/off switches, phenotypes often vary along a continuous scale or graded spectrum, reflecting differences in the strength of the underlying biological output [16]. An intuitive analogy is a rheostat in an electronic circuit, where the final output of the system depends on the coordinated and cooperative contributions of multiple adjustable control elements.

Methodologically, this computational approach is closely related to differential co-expression/differential correlation analyses, which detect the “rewiring” of gene–gene relationships across conditions [11,12]. Regulostat Inferelator (RSI) focuses specifically on identifying gene pairs whose associated strength and/or directionality changes with quantitative phenotypes (e.g., drug response). We used the Cancer Cell Line Encyclopedia (CCLE) as a proof-of-principle study. RSI operationalizes each gene–gene co-expression relationship (e.g., correlation coefficient) as a rheostat-like unit and identifies gene pairs whose systematic change tracks phenotype variation. In doing so, RSI highlights candidate gene–gene interactions that may be actionable “control points” for phenotypic engineering to alter response phenotypes.

#### 2.2.3. Weight-Engineered Gene Associations

Artificial Neural Networks (ANNs) have gained much popularity for their power to learn various tasks, from inferring disease diagnosis to the generation of novel protein sequences. However, a critical question is whether the “knowledge” learned by a trained ANN model can be decoded and used to explain why certain associations between biological entities (i.e., genes) contribute to the predictive performance of trained models. Although the workings of ANNs are often regarded as a black box, we proposed that inter-neuronal weights of the ANN learned during the training phase encode valuable “knowledge”.

In our view, an ANN model acts as a “little brain”. Similarly to the human brain, which stores and processes information through patterns of activity and synaptic weights (i.e., strength) between neurons, an ANN learns by adjusting the weights across interconnected neurons. Using this approach, we developed weight engineering, which is a neural network-based reverse engineering approach to extract meaningful associations between data attributes and decode embedded knowledge learned by an ANN model [27]. We developed an Artificial Neural Network Encoder (ANNE), which uses an autoencoder architecture and implements weight engineering to decode nonlinear relations between genes under a specific biological state. We showcase this by using a breast cancer dataset where patients were subjected to neoadjuvant taxane–anthracycline chemotherapy and for whom a prognosis was available. Patients were stratified according to pathologic complete response (pCR, i.e., no invasive or metastatic cancer identified) and chemoresistance, defined by the residual burden (RB, i.e., early relapses). Models for prognostic outcomes were defined using distant relapse-free survival (DRFS). Both drug response and prognostic models were trained using ANNE. Once trained, we employed our weight engineering approach to reverse engineer gene–gene associations, where each node represents a gene, and gene–gene interactions are defined by the strength of learned weights. These learned gene–gene associations were further aggregated to derive disease networks that aim to explain clinical traits underlying the disease. Our work shows that it is possible to open the black box of ANN models and extract the learned knowledge via weight engineering to uncover hidden patterns, i.e., gene–gene associations that explain the behavior of a disease phenotype.

#### 2.2.4. Gene Utility

Since every gene interacts with one another in a cellular PPI network to exert its function, those mutated and differentially expressed genes are not necessarily the most important genes in conferring and sustaining a disease state. We recently proposed the concept of the Gene Utility Model (GUM) [8], which states that how a gene is used in the PPI network under a specific disease context determines its importance to disease development. Unlike genetic mutations and differential gene expressions that can be detected and measured experimentally, gene utility is a more abstract concept to grasp, as it cannot be directly observed and quantified by experimental means. Thus, gene utility is a latent property of genes. However, one can perceive gene utility as how frequently a protein interacts with its counterparts in a PPI network to exert different functions in cells. A protein that undergoes rapid degradation and has a small number of interactions with its interacting counterparts is deemed low utility and, hence, is less important to the disease state. Formally, the utility of a gene can be recapitulated as the amount of information that flows through the PPI network. Using a process-guided flow algorithm, implemented in a tool like NetDecoder, we can model information flows as a proxy for gene utility [28].

We evaluated GUM in neuroblastoma, a childhood cancer that accounts for ~8–10% of pediatric tumors, which is often metastatic, and has a <40% 5-year survival in high-risk disease [29]. Neuroblastoma is characterized by fewer somatic mutations than many adult cancers [15] but exhibits recurrent chromosomal aberrations (e.g., 1p loss of heterozygosity and 17q gain) that are associated with MYCN amplification (2p24). The mechanistic basis for this conserved karyotype remains unclear. Another intriguing feature of NB is the frequent overexpression of TP53 despite TP53 being a canonical tumor suppressor in many cancers [30]. Using GUM, we found that genes located on the 1p and 17q arms are, respectively, lowly utilized and highly utilized, suggesting an evolutionary rationale for why these aberrations are conserved. GUM also indicated low utility of TP53, which is consistent with the idea that TP53’s tumor-suppressive role may be attenuated in neuroblastoma despite its high expression. These results suggest that gene utility not only can complement mutational and expression profiles, but it is a more reliable indicator for prioritizing disease-relevant genes.

#### 2.2.5. Symmetric Relationships of Gene Expression

Complex phenotypes can be remarkably stable at both cellular and organismal levels despite substantial inter-individual genetic heterogeneity. This motivated our search for invariant relationships in molecular readouts. We hypothesize that if a phenotype can be stably maintained across many genetic backgrounds, then, according to the principle of symmetry, there exist transcriptomic patterns that are invariant (i.e., unchanged) within that phenotype. In mathematics and physics, such invariances are often described as features of symmetry, a property that remains stable (or invariant) upon a particular operation (e.g., reflection, rotation, and translation). Here, we borrow the language of symmetry to formalize phenotype-invariant gene-expression relationships.

In this context, we propose the Gene Expression Symmetry Hypothesis (GESH), which treats genetic variations among individuals as a set of perturbations (i.e., “transformations”) that are applied to the underlying molecular system. GESH posits that if a cohort of individuals shares the same phenotype, these perturbations do not substantially alter gene expression relationships of certain gene pairs that remain approximately invariant within that phenotype. Gene pairs that satisfy this criterion are defined as Invariant Feature Genes (IFGs).

We recently devised Learning-Based Invariant Feature Engineering (LIFE) [11], which is a hybrid machine learning approach that operationalizes the concept of invariance of gene expression. We devised the following two symmetry-preserving functions, called Invariant Feature Functions (IFFs), to identify IFGs:

$$IFF1=\frac{g_{i}}{g_{j}}+\frac{g_{j}}{g_{i}}\;\sim \;constant\;and\;IFF2=g_{i}\cdot g_{j}\;\sim \;constant$$

where $g_{i}$ and $g_{j}$ are the expression levels of gene-*i* and gene-*j*, respectively, in a sample for a given biological phenotype. Both *IFF*1 and *IFF*2 describe symmetric expression relations between genes $g_{i}$ and $g_{j}$ since the subscripts *i* and *j* can be interchanged without altering the computation results. *IFF*2 denotes the multiplication of the expression values of gene-*i* and gene-*j*, not the vector-based dot product operation. Pairs of gene-*i* and gene-*j* whose expression yields invariant single outputs (i.e., small standard deviations) across samples of the same phenotype upon transformation by *IFF*1 or *IFF*2 are considered IFG candidates.

Using transcriptomics data derived from 25 types of normal organs and 25 cancer types as a proof-of-concept study, we employed multiclass classification to assess the performance of IFGs in classifying samples into their respective phenotypes. For each biological phenotype, we selected the top 1000 gene pairs with the smallest standard deviations with respect to *IFF*1 (IF1_1000_) and *IFF*2 (IF2_1000_) being IFG candidates. Our multiclass classification results show that IFGs are indeed phenotype-specific fingerprints that can accurately classify individual samples to their respective organs or cancer types. Furthermore, we demonstrated that the expression values of hubs for IFG-constructed networks (IF-Nets) can be used to reconstruct sample-wise expression values in relation to their counterpart genes, meaning these hubs are information encoders. These symmetry-based layers can enrich the MGM by highlighting gene relationships that persist across genetic heterogeneity and may, therefore, point to core regulatory constraints of the phenotype.

#### 2.2.6. Functional Latent Genetic Mutations

A driver genetic mutation confers a growth or survival advantage that is typically identified through statistical evidence of positive selection, such as recurrent mutation hotspots occurring at rates exceeding the expected background of a neutral passenger [31,32]. Nonetheless, recurrent mutations are uncommon, and mutation frequencies frequently follow a long-tail distribution [15,33], making it challenging to pinpoint functional driver mutations that do not meet statistical criteria. Raphael and colleagues previously developed HotNet, a network-based algorithm that implements the idea of heat diffuse/propagate over the PPI network to score mutations that significantly alter gene subnetworks [34,35]. Similarly, Hofree et al. developed Network-Based Stratification (NBS) that uses a network propagation algorithm to “smooth” mutations over a disease network to cluster tumors into subtypes [36], and Paul et al. developed TieDIE (diffusion linking genomic to transcriptomic states) that employs a diffusion-based approach to connect upstream genomic perturbations to downstream expression programs via interaction networks [37]. We previously devised PERMUTOR (PERsonalized MUtation evaluaTOR), which implements the principle of least action on individual patients’ tumor mutations and omics context and builds a patient-specific “disease module” network (a subnetwork of the interactome) de novo [38]. PERMUTOR provides scores where mutated genes are the most important for a specific patient and predicts the types of therapeutic target genes (single and/or combinations) that can significantly disrupt a patient’s disease module. All these computational methods have the power to detect functional latent genetic mutations that escape conventional statistical tests.

## 3. Building a Disease-Specific Mini-Galaxy Model

**Figure 3:**
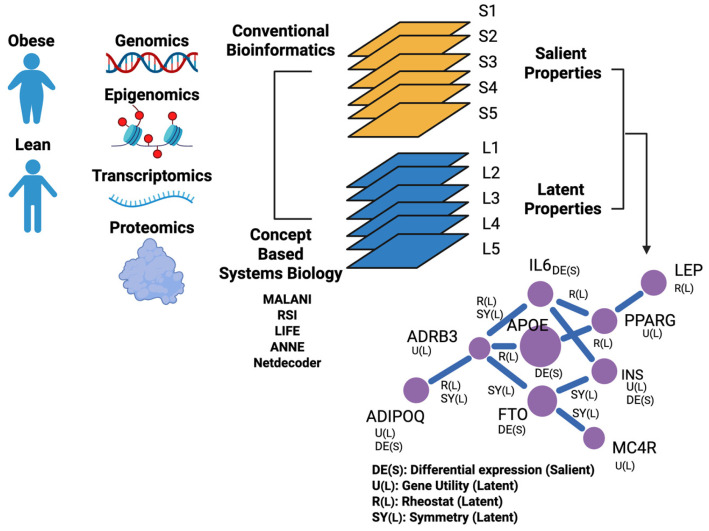
Building a disease-specific Mini-Galaxy Model (illustrated in obesity). Multi-omics data (e.g., genomics, epigenomics, transcriptomics) from lean and obese individuals can be collected from public repositories. Under the MGM, genes can exhibit multiple properties—salient and latent—each represented as an information layer. Conventional comparative bioinformatics pipelines identify salient characteristics associated with obesity, whereas hypothesis-guided systems biology and AI algorithms infer latent characteristics. A disease-specific MGM can be constructed as a multilayer gene network by superimposing these layers (or by maintaining them as a multiplex network), where nodes are genes and edges represent layer-specific gene–gene associations. This multilayer representation supports downstream analyses to nominate multilayer hubs and pathways that are consistently implicated across evidence types. Genes shown in the network at the bottom right are known as obese-associated genes, but their relations are hypothetical and only displayed for illustrative purposes. (Created in BioRender. Li, H. (2026) https://BioRender.com/ch0euqc).

We use obesity as an illustrative example for constructing a disease-specific MGM. First, multi-omics (genomics, GWAS, epigenomics, transcriptomics, etc.) generated in lean and obese individuals can be retrieved from public repositories such as Gene Expression Omnibus [39], GTEx, and EMBL-EBI Expression Atlas [40]. Salient modes of gene characteristics in obesity can be identified by conventional bioinformatics and systems biology analysis tools [41,42,43,44]. In contrast, latent gene characteristics that modulate obesity are not directly measurable and require phenotype-linked inference (Figure 3). For instance, MALANI [10] is used to infer “dark” gene associations that drive obesity; RSI [9] is used to detect gene pairs that are acting as rheostat-like switches that “flip” between lean and obesity contexts; ANNE, which implements a weight engineering algorithm [27], is used to reverse engineer associations between genes that explain clinical traits of obesity; NetDecoder [28] is used to identify highly utilized genes in obese patients; and LIFE [11] is used to uncover gene pairs that exhibit symmetric expressions that served as molecular “fingerprints” that define lean against obese traits.

Next, each set of salient and latent gene characteristics is used to build an information layer. These information layers, and their respective gene–gene interaction networks, are then superimposed to create an integrated multimodal network that we call the “mini-galaxy” in a disease. Here, we use obesity as our illustrative example. As shown in Figure 3, a disease-specific MGM is a graph that comprises nodes that represent genes and edges that denote gene–gene associations. Unlike conventional biological networks or knowledge graphs, disease-specific MGMs capture multiple latent gene properties that are not accounted for by direct observation and experimental measurements. In an MGM, each node and edge contain multimode information on genes and their associations.

### 3.1. Synthetizing Multilayered Information into an MGM

Consider a hypothetical MGM with nine frequently reported obese-associated genes [45,46], as shown in the lower right panel of Figure 3. Suppose a salient differential expression layer flags APOE and IL6 as being altered between obese and lean adipocytes, while a co-expression layer includes edges PPARG-LEP, PPARG-IL6, and PPARG-APOE. Based on regulostat inference, a gene utility layer could assign higher utility to PPARG if it lies on many adipogenesis information-flow paths. When superimposed, PPARG connects across multiple layers and emerges as a candidate for a multilayer hub for follow-up experimentation.

After defining salient and latent properties, each layer can be represented as a gene–gene network (an adjacency matrix) with a consistent node set (genes). Layers will differ in edge semantics (e.g., co-expression correlation, inferred “dark” association, flow-based coupling), but can be scaled and normalized for comparison to enable integration. A disease-specific MGM can then be represented as (i) a multiplex network that preserves layer identities for layer-aware analyses (e.g., multilayer centrality), (ii) an aggregated network using layer-specific weights, or (iii) a multi-relational graph that is amenable to representation learning to build interpretable machine learning models. These design choices influence interpretability, robustness, and scalability, and should be reported explicitly to support reproducibility and cross-study comparisons.

### 3.2. Practical Considerations for MGM Construction and Validation

*Layer definition and normalization*. Each MGM layer should specify (i) the node set (e.g., genes mapped to consistent identifiers), (ii) the edge semantics (what an edge means biologically and how it was inferred), and (iii) an edge-weighted scale. Because layers can be generated by heterogeneous algorithms (e.g., correlations, SVM-inferred interactions, information flow), edge weights should be normalized (e.g., z-scores or rank-based scaling) before integration so that no single layer dominates simply due to its numeric range. When possible, layer-specific confidence estimates (e.g., cross-validated stability of an edge) can be retained as weights to improve robustness.

*Operational definition of multimode hubs*. To move beyond the metaphor of “gravitational centers”, we define a multilayer information hub as a gene that is central across multiple layers (salient and latent) after layer normalization. Centrality measures (e.g., degree of connectivity and betweenness) of a gene in each layer will be normalized within a layer (e.g., z-score). A multimode hub score for a gene of interest can then be computed by aggregating connectivity across layers that represent different gene actions, and by combining the number of cross-layer links with corresponding edge weights to capture both the breadth and strength of evidence. This multimode hub score not only indicates the overall importance of a hub gene but also reveals the specific gene actions for a given hub gene that contribute to a disease-specific MGM.

*Layer weighting, regularization, and model selection*. One practical challenge is determining how much influence each layer should have in downstream analyses. Weights can be set heuristically (e.g., equal weights), learned from data using a downstream objective (e.g., phenotype prediction), or selected based on stability across resampling. Because the MGM integrates many features, guarding against overfitting is essential; nested cross-validation, independent validation cohorts, and explicit reporting of model-selection procedures are recommended by following best-practice guidance for machine learning in genomics [47]. Regularization strategies, including using sparsity-inducing approaches in each layer, penalizing redundant layers, or using stability selection to retain edges that are reproducibly detected, can improve generalization and interpretability. Layer-aware integration strategies from multi-omics learning and factor models can also inform these design choices [48,49].

*Layer compatibility, redundancy, and uncertainty*. The MGM does not assume that all latent layers are equally informative or mutually compatible. In practice, layers can be redundant (e.g., high edge overlap or highly correlated centrality profiles) or noisy (e.g., false positives from different inference methods). Uncertainty from one layer might propagate to other layers. We therefore recommend (i) reporting the hypothesis that generates the outputs and data requirements for each layer, (ii) developing scores to quantify redundancy (e.g., edge overlap, correlation of node scores, or agreement of enriched pathways), and (iii) removing layers with low reliability.

*Sensitivity to data quality, cohort size, and batch effects*. Several latent layers are inferred from phenotype-linked omics (e.g., gene utility) and are therefore sensitive to confounding (e.g., batch, platform, and other hidden covariates such as age and sex) and a limited sample size. For each data modality, we recommend explicit quality control, appropriate normalization (as mentioned above), and confounder correction (e.g., including known covariates, surrogate-variable approaches, or batch-harmonization) prior to layer inference. Additionally, layer stability can be assessed with resampling and, when possible, by replication across cohorts to reduce noise propagation into the integrated MGM.

*Scalability*. Naïvely constructing many dense gene–gene layers can be computationally expensive. Practical strategies include restricting analyses to phenotype-relevant gene sets (e.g., expressed genes in a tissue, GWAS-implicated loci, or pathway-defined modules), sparsifying edges by using statistically justified thresholds, and parallelizing layer construction. For large cohorts, approximate algorithms (e.g., sampling-based correlation estimation, randomized singular value decomposition (SVD) for embeddings) can reduce runtime while preserving key structures.

*Reproducibility and benchmarking roadmap*. To achieve MGM results that are comparable across studies, it is important to report the following: (i) data sources and preprocessing methods, (ii) the full definition of each layer (algorithm, parameters, thresholds), and (iii) describe how the layers were integrated. Containerized workflows and versioned code releases can further improve reproducibility. For validation, MGM-derived hubs and pathways can be benchmarked against curated disease genes, pathway databases, and drug-target resources, and compared with baselines such as single-layer PPI networks, multi-omics integration methods (e.g., similarity network fusion) [48], and knowledge-graph-based approaches [12,50]. Multilayer network theory provides additional metrics (e.g., multilayer centrality) that can be used to quantify the added value of preserving layer structure rather than collapsing all evidence into a single graph.

## 4. What Mini-Galaxy Models Reveal

An MGM is built for a phenotype of interest by integrating salient and latent gene properties and using the strategies outlined in the previous sections. However, building networks with multilayered information is not new. For instance, Buphamalai et al. built a multiplex network with many layers spanning biological scales, connecting genotype-to-phenotype via layer-wise gene relationships [51]. The work used a cross-layer structure to interpret mechanisms and predict disease genes. What is novel for the MGM is the incorporation of latent gene properties (like hidden matter in a galaxy) into multilayered biological networks to uncover hidden modes of gene actions in driving disease development. The subsequent analysis is very much akin to conventional network analysis [5,52] but with a few modifications.

**Figure 4:**
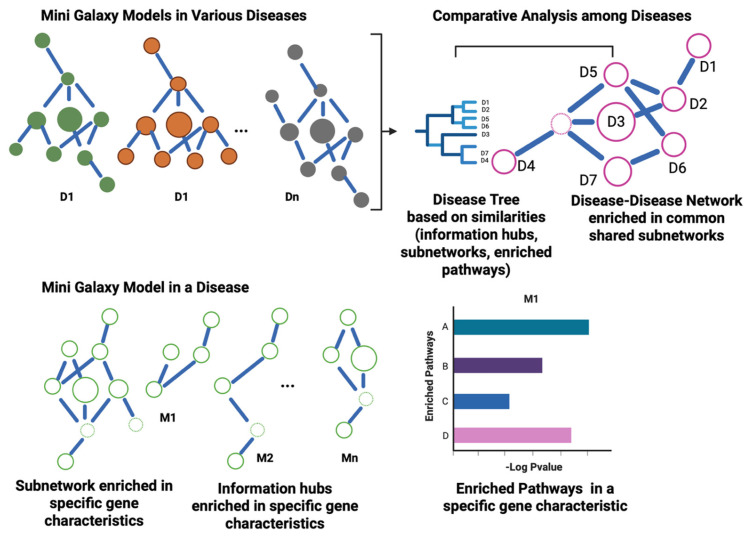
Translating Mini-Galaxy Models into clinical insights. A Mini-Galaxy Model (MGM) can be built for each disease. Within a disease, network enrichment methods can be employed to identify subnetworks enriched with specific salient and latent gene characteristics. The size of the enriched subnetwork reflects the importance of that type of property. Prioritized hubs detected in both salient and latent layers rank the highest. Similarly, biological pathways enriched across multilayers carry greater disease importance. For MGM comparative analysis across diseases (D1 to D7), distance-based metrics can be employed to determine network similarities involving information hubs, salient and latent specific subnetworks, and pathways among MGMs. Disease hierarchies can be inferred using similarity measures (e.g., information hubs) or overall similarity (i.e., information hubs + mode-specific subnetworks + mode-enriched pathways) to define relationships between diseases. Alternatively, disease–disease network comparisons can be constructed using similarity metrics such as the Jaccard Index. (Created in BioRender. Li, H. (2026) https://BioRender.com/oc4klhn).

The goal is to extract the following information embedded in a disease-specific MGM: (i) which genes of the salient and latent modes play major roles, (ii) defining what genes act as information hubs in a given mode; (iii) discovering what genes are information hubs across multiple modes, and (iv) assessing biological pathways that are enriched in a set of salient and latent characteristics or enriched across multiple characteristics. The defined approach can be performed within a disease type and across distinct diseases (Figure 4).

### 4.1. Analysis Within a Disease Type

For analysis within a disease type, we aim to uncover which salient or latent gene properties are disease-specific. The underlying hypothesis is that genes showing a multimode property with high degrees of connectivity in the MGM are gravitational centers—information hubs—and focal points in the disease and, as such, can be used to rank importance. Using network-based enrichment analysis approaches such as EnrichNet [53] and NetPEA [54], we can extract key subnetworks enriched in a disease.

### 4.2. Comparative Analysis Between Diseases

Knowing how different diseases relate to one another enhances our understanding of common mechanisms and can aid the design of novel strategies to improve patient care, formulate novel treatment designs, and define routes for disease prevention. “Link by similarity” is the underlying idea. By comparing Mini-Galaxy Models (MGMs) between distinct diseases, we can define similarities. One strategy is to use a distance-based metric to quantify similarities between diseases, as in hierarchical clustering [55]. The resulting dendrogram defines a “disease tree”, which is analogous to a phylogenetic tree, where clades illustrate evolutionary relationships. A disease tree can integrate the similarity measures between salient and latent gene properties, specific information hubs, and shared enriched pathways. Diseases that are grouped under the same “clade” share conserved disease-driving mechanisms. Alternatively, network-based similarity scores, such as the Jaccard Index [47], can also be used to assess similarity between MGMs across distinct diseases. Another strategy is to use pairwise comparisons to uncover relationships between diseases and then summarize them in a differential disease network.

## 5. Mini-Galaxy Model as a Discovery Platform for Translational Medicine

**Figure 5:**
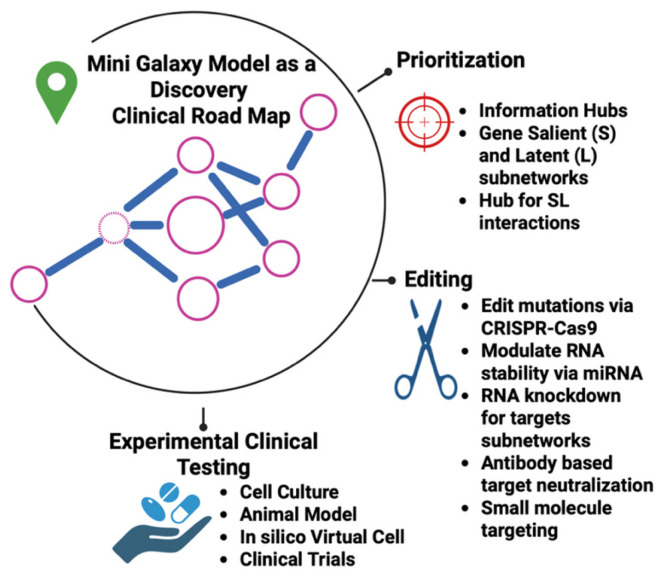
The Mini-Galaxy Model as a discovery platform. The Mini-Galaxy framework identifies and ranks salient and latent properties across multiple biological scales, offering a road map to clinical investigation. Top-ranked genes, which capture information hubs across prioritized salient and latent layers, are drivers of disease. These genes can be manipulated by using gene editing approaches, RNA modulation via miRNAs or RNAi knockdown, and inhibitors like antibody-based or small molecular drugs, and are followed up in cell culture, organoids, and mouse models. The MGM also offers an in silico virtual cell modeling framework to simulate the effect of perturbations. Prioritized and pre-clinical validated targets are potential targets for clinical trials. (Created in BioRender. Li, H. (2026) https://BioRender.com/8b0kpmf).

Since one MGM integrates multiple gene information layers, it can be used to inform novel medical discoveries. It can be particularly valuable for target prioritization and gene editing, followed by experimental and pre-clinical testing (Figure 5). Gene editing involves, for example, using CRISPR-Cas9 editing for genetic mutations and manipulation of miRNAs or RNAi knockdowns, or through protein inhibitions by antibody-based or small molecular drugs. For instance, we have shown that hubs in derided cancer IF-Nets are enriched with both approved and clinical trial drugs, highlighting that “symmetry breaking” is a novel approach to disrupt diseases [11]. Our previous work, using Gene Utility Model (GUM), had identified the tau kinase FYN and the E3 ubiquitin ligase as key players in tauopathy in PS19 mice. Strikingly, we also identified proto-oncogene/transcription factor MYC, the SUMO-conjugating enzyme UBE2I (Ubc9), and the EGF receptor (EGFR) as important players. Our experimental pharmacological inhibition of either MYC or EGFR confirmed protection against tau toxicity [56], indicating that intervening gene utilities can result in pharmacological benefits.

The clinical relevance of MGMs can be evaluated in pre-clinical settings, including studies with cells, organoids, micro-cancer, and animal models. The goal is to assess to what extent intervening prioritized multimodal-displaying genes can ameliorate a disease phenotype, like restoring cell morphological phenotypes, homeostasis of cell–cell communications, or resolution of disease lesions. Multi-omics data can be generated to confirm changes after interventions. In addition, in silico virtual cell models [57,58] can also be employed to simulate the effects of biological editing. Finally, once the prioritized targets have passed all experimental and computational validations, they can advance into clinical trials.

## 6. Conclusions

In conclusion, we propose the Mini-Galaxy Model (MGM), in which cells are conceptualized as a “mini-galaxy”. The MGM is a systems-based, multimode framework that unifies salient (mutations, epigenetic modifications, differential gene expression) and latent (gene utility, symmetric gene expression, dark gene associations, regulostat-like gene–gene associations, and weight engineering-derived gene–gene relations) gene characteristics into a single, disease-specific integrative map. By treating genes that integrate multiple informational layers as “information hubs,” the MGM reframes disease as the emergent outcome across these layers. This framework will enable rigorous analyses within and across diseases to identify dominant gene characteristics, recover informational hub genes, and pinpoint multimode pathways that collectively drive pathology. Thus, the MGM provides the structure needed to convert heterogeneous omics into coherent, mechanism-grounded hypotheses. It further situates diseases to be placed in a shared landscape, leveraging multimode similarity.

We also discussed how an MGM can serve as a discovery platform to promote translational medicine, in particular by offering a prioritization strategy and follow-up steps. This innovation goes beyond target-discovery methods, which usually focus only on a few gene features, such as genetic variants, mutations, epigenetics, and differential expressions. Importantly, incorporating multiple layers of gene behaviors also opens new possibilities for repurposing existing drugs. In a recent study, we found that many FDA-approved drugs align with key hub genes in a network enriched for symmetric gene-expression modes [11]. We also anticipate that multimode interventions will enhance therapeutic efficacy and reduce drug toxicity effects, while preserving target specificity by engaging multiple genes.

In short, the MGM provides a new conceptual framework for modeling disease and consolidates a strategy to aggregate heterogeneous omics into an integrative, mechanism-grounded map that streamlines target selection, defines a rationale for drug combinations and drug repurposing, and overall, provides a general blueprint for moving from AI network system insights into clinical translation.

## Data Availability

All data are publicly available.

## Abbreviations

The following abbreviations are used in this manuscript:

MGM	Mini-galaxy model
PPI	Protein–Protein interactome
AI	Artificial intelligence
GWAS	Genome-wide association study
pCR	Pathological complete response
DRFS	Distant relapse-free survival
ANN	Artificial neural network
SVM	Support vector machines

## References

[1] Ung C.Y., Correia C., Billadeau D.D., Zhu S., Li H. (2023). Manifold epigenetics: A conceptual model that guides engineering strategies to improve whole-body regenerative health. Front. Cell Dev. Biol..

[2] Ung C.Y., Weiskittel T.M., Correia C., Kaufmann S.H., Li H. (2022). Manifold medicine: A schema that expands treatment dimensionality. Drug Discov. Today.

[3] Ung C.Y., Correia C., Li H., Adams C.M., Westendorf J.J., Zhu S. (2024). Multiorgan locked-state model of chronic diseases and systems pharmacology opportunities. Drug Discov. Today.

[4] Castro-Giner F., Ratcliffe P., Tomlinson I. (2015). The mini-driver model of polygenic cancer evolution. Nat. Rev. Cancer.

[5] Barabasi A.L., Gulbahce N., Loscalzo J. (2011). Network medicine: A network-based approach to human disease. Nat. Rev. Genet..

[6] Vidal M., Cusick M.E., Barabasi A.L. (2011). Interactome networks and human disease. Cell.

[7] Menche J., Sharma A., Kitsak M., Ghiassian S.D., Vidal M., Loscalzo J., Barabasi A.L. (2015). Disease networks. Uncovering disease-disease relationships through the incomplete interactome. Science.

[8] Ung C.Y., Levee T.M., Zhang C., Correia C., Yeo K.S., Li H., Zhu S. (2022). Gene utility recapitulates chromosomal aberrancies in advanced stage neuroblastoma. Comput. Struct. Biotechnol. J..

[9] Ung C.Y., Ghanat Bari M., Zhang C., Liang J., Correia C., Li H. (2019). Regulostat Inferelator: A novel network biology platform to uncover molecular devices that predetermine cellular response phenotypes. Nucleic Acids Res..

[10] Ghanat Bari M., Ung C.Y., Zhang C., Zhu S., Li H. (2017). Machine Learning-Assisted Network Inference Approach to Identify a New Class of Genes that Coordinate the Functionality of Cancer Networks. Sci. Rep..

[11] Zhang C., Correia C., Weiskittel T., Tan S.H., Zhang Z., Yeo K.S., Zhu S., Ung C.Y., Li H. (2025). Symmetry as a Fundamental Principle in Defining Gene Expression and Phenotypic Traits. bioRxiv.

[12] Li M.M., Huang K., Zitnik M. (2022). Graph representation learning in biomedicine and healthcare. Nat. Biomed. Eng..

[13] Richards S., Aziz N., Bale S., Bick D., Das S., Gastier-Foster J., Grody W.W., Hegde M., Lyon E., Spector E. (2015). Standards and guidelines for the interpretation of sequence variants: A joint consensus recommendation of the American College of Medical Genetics and Genomics and the Association for Molecular Pathology. Genet. Med..

[14] Mohiuddin M., Kooy R.F., Pearson C.E. (2022). De novo mutations, genetic mosaicism and human disease. Front. Genet..

[15] Vogelstein B., Papadopoulos N., Velculescu V.E., Zhou S., Diaz L.A., Kinzler K.W. (2013). Cancer genome landscapes. Science.

[16] Jones P.A. (2012). Functions of DNA methylation: Islands, start sites, gene bodies and beyond. Nat. Rev. Genet..

[17] Strahl B.D., Allis C.D. (2000). The language of covalent histone modifications. Nature.

[18] Shang R., Lee S., Senavirathne G., Lai E.C. (2023). microRNAs in action: Biogenesis, function and regulation. Nat. Rev. Genet..

[19] Hentze M.W., Castello A., Schwarzl T., Preiss T. (2018). A brave new world of RNA-binding proteins. Nat. Rev. Mol. Cell Biol..

[20] de Leeuw C.A., Neale B.M., Heskes T., Posthuma D. (2016). The statistical properties of gene-set analysis. Nat. Rev. Genet..

[21] Letourneau A., Santoni F.A., Bonilla X., Sailani M.R., Gonzalez D., Kind J., Chevalier C., Thurman R., Sandstrom R.S., Hibaoui Y. (2014). Domains of genome-wide gene expression dysregulation in Down’s syndrome. Nature.

[22] Rosati D., Palmieri M., Brunelli G., Morrione A., Iannelli F., Frullanti E., Giordano A. (2024). Differential gene expression analysis pipelines and bioinformatic tools for the identification of specific biomarkers: A review. Comput. Struct. Biotechnol. J..

[23] Kohler S., Bauer S., Horn D., Robinson P.N. (2008). Walking the interactome for prioritization of candidate disease genes. Am. J. Hum. Genet..

[24] Vanunu O., Magger O., Ruppin E., Shlomi T., Sharan R. (2010). Associating genes and protein complexes with disease via network propagation. PLoS Comput. Biol..

[25] Ghiassian S.D., Menche J., Barabasi A.L. (2015). A DIseAse MOdule Detection (DIAMOnD) algorithm derived from a systematic analysis of connectivity patterns of disease proteins in the human interactome. PLoS Comput. Biol..

[26] Novakovsky G., Dexter N., Libbrecht M.W., Wasserman W.W., Mostafavi S. (2023). Obtaining genetics insights from deep learning via explainable artificial intelligence. Nat. Rev. Genet..

[27] Zhang C., Correia C., Weiskittel T.M., Tan S.H., Meng-Lin K., Yu G.T., Yao J., Yeo K.S., Zhu S., Ung C.Y. (2022). A Knowledge-Based Discovery Approach Couples Artificial Neural Networks With Weight Engineering to Uncover Immune-Related Processes Underpinning Clinical Traits of Breast Cancer. Front. Immunol..

[28] da Rocha E.L., Ung C.Y., McGehee C.D., Correia C., Li H. (2016). NetDecoder: A network biology platform that decodes context-specific biological networks and gene activities. Nucleic Acids Res..

[29] Maris J.M. (2010). Recent advances in neuroblastoma. N. Engl. J. Med..

[30] Van Maerken T., Vandesompele J., Rihani A., De Paepe A., Speleman F. (2009). Escape from p53-mediated tumor surveillance in neuroblastoma: Switching off the p14(ARF)-MDM2-p53 axis. Cell Death Differ..

[31] Dees N.D., Zhang Q., Kandoth C., Wendl M.C., Schierding W., Koboldt D.C., Mooney T.B., Callaway M.B., Dooling D., Mardis E.R. (2012). MuSiC: Identifying mutational significance in cancer genomes. Genome Res..

[32] Martincorena I., Raine K.M., Gerstung M., Dawson K.J., Haase K., Van Loo P., Davies H., Stratton M.R., Campbell P.J. (2017). Universal Patterns of Selection in Cancer and Somatic Tissues. Cell.

[33] Salk J.J., Fox E.J., Loeb L.A. (2010). Mutational heterogeneity in human cancers: Origin and consequences. Annu. Rev. Pathol..

[34] Vandin F., Upfal E., Raphael B.J. (2011). Algorithms for detecting significantly mutated pathways in cancer. J. Comput. Biol..

[35] Vandin F., Clay P., Upfal E., Raphael B.J. (2012). Discovery of mutated subnetworks associated with clinical data in cancer. Pac. Symp. Biocomput..

[36] Hofree M., Shen J.P., Carter H., Gross A., Ideker T. (2013). Network-based stratification of tumor mutations. Nat. Methods.

[37] Paull E.O., Carlin D.E., Niepel M., Sorger P.K., Haussler D., Stuart J.M. (2013). Discovering causal pathways linking genomic events to transcriptional states using Tied Diffusion Through Interacting Events (TieDIE). Bioinformatics.

[38] Weiskittel T.M., Ung C.Y., Correia C., Zhang C., Li H. (2022). De novo individualized disease modules reveal the synthetic penetrance of genes and inform personalized treatment regimens. Genome Res..

[39] Barrett T., Wilhite S.E., Ledoux P., Evangelista C., Kim I.F., Tomashevsky M., Marshall K.A., Phillippy K.H., Sherman P.M., Holko M. (2013). NCBI GEO: Archive for functional genomics data sets--update. Nucleic Acids Res..

[40] Papatheodorou I., Fonseca N.A., Keays M., Tang Y.A., Barrera E., Bazant W., Burke M., Fullgrabe A., Fuentes A.M., George N. (2018). Expression Atlas: Gene and protein expression across multiple studies and organisms. Nucleic Acids Res..

[41] Perez G., Barber G.P., Benet-Pages A., Casper J., Clawson H., Diekhans M., Fischer C., Gonzalez J.N., Hinrichs A.S., Lee C.M. (2025). The UCSC Genome Browser database: 2025 update. Nucleic Acids Res..

[42] Koboldt D.C., Zhang Q., Larson D.E., Shen D., McLellan M.D., Lin L., Miller C.A., Mardis E.R., Ding L., Wilson R.K. (2012). VarScan 2: Somatic mutation and copy number alteration discovery in cancer by exome sequencing. Genome Res..

[43] Love M.I., Huber W., Anders S. (2014). Moderated estimation of fold change and dispersion for RNA-seq data with DESeq2. Genome Biol..

[44] Robinson M.D., McCarthy D.J., Smyth G.K. (2010). edgeR: A Bioconductor package for differential expression analysis of digital gene expression data. Bioinformatics.

[45] Loos R.J.F., Yeo G.S.H. (2022). The genetics of obesity: From discovery to biology. Nat. Rev. Genet..

[46] Banerjee D., Girirajan S. (2025). Discovery of obesity genes through cross-ancestry analysis. Nat. Commun..

[47] Fuxman Bass J.I., Diallo A., Nelson J., Soto J.M., Myers C.L., Walhout A.J. (2013). Using networks to measure similarity between genes: Association index selection. Nat. Methods.

[48] Wang B., Mezlini A.M., Demir F., Fiume M., Tu Z., Brudno M., Haibe-Kains B., Goldenberg A. (2014). Similarity network fusion for aggregating data types on a genomic scale. Nat. Methods.

[49] Argelaguet R., Velten B., Arnol D., Dietrich S., Zenz T., Marioni J.C., Buettner F., Huber W., Stegle O. (2018). Multi-Omics Factor Analysis-a framework for unsupervised integration of multi-omics data sets. Mol. Syst. Biol..

[50] Himmelstein D.S., Lizee A., Hessler C., Brueggeman L., Chen S.L., Hadley D., Green A., Khankhanian P., Baranzini S.E. (2017). Systematic integration of biomedical knowledge prioritizes drugs for repurposing. eLife.

[51] Buphamalai P., Kokotovic T., Nagy V., Menche J. (2021). Network analysis reveals rare disease signatures across multiple levels of biological organization. Nat. Commun..

[52] Goh K.I., Cusick M.E., Valle D., Childs B., Vidal M., Barabasi A.L. (2007). The human disease network. Proc. Natl. Acad. Sci. USA.

[53] Glaab E., Baudot A., Krasnogor N., Schneider R., Valencia A. (2012). EnrichNet: Network-based gene set enrichment analysis. Bioinformatics.

[54] Liu L., Ruan J. Network-based Pathway Enrichment Analysis. Proceedings of the IEEE International Conference on Bioinformatics and Biomedicine.

[55] Eisen M.B., Spellman P.T., Brown P.O., Botstein D. (1998). Cluster analysis and display of genome-wide expression patterns. Proc. Natl. Acad. Sci. USA.

[56] Apicco D.J., Zhang C., Maziuk B., Jiang L., Ballance H.I., Boudeau S., Ung C., Li H., Wolozin B. (2019). Dysregulation of RNA Splicing in Tauopathies. Cell Rep..

[57] Bunne C., Roohani Y., Rosen Y., Gupta A., Zhang X., Roed M., Alexandrov T., AlQuraishi M., Brennan P., Burkhardt D.B. (2024). How to build the virtual cell with artificial intelligence: Priorities and opportunities. Cell.

[58] Johnson G.T., Agmon E., Akamatsu M., Lundberg E., Lyons B., Ouyang W., Quintero-Carmona O.A., Riel-Mehan M., Rafelski S., Horwitz R. (2023). Building the next generation of virtual cells to understand cellular biology. Biophys. J..

